# Dangers in Neck Wear

**Published:** 1917-12

**Authors:** 


**Dangers in Neck Wear.** By Walter G. Walford, M.D. Pp. x. 171. Illustrated. London : H. K. Lewis & Co. Ltd. 1917. Price 4s. 6d. net.

One might have felt impressed with the dangers the author refers to had he not allowed his observations to lead him to

[1] The Secretary informs us that, in page 278, " there is one matter in the History which appears to me to give a totally wrong impression : That the Bristol General Hospital was founded owing to our inability to provide sufficient beds. In the Smith papers there is preserved the correspondence in the papers and an apology from the promotors of the Bristol General Hospital, in which it is clearly stated that if the Infirmary did not unduly oppose the establishment of a second institution they on their part would take care never in any way to compete with or injure the B.R.I."

an obsession. When we are told of a case of pernicious anæmia caused by tight neck wear, and that it is not unreasonable to think that diabetes may be attributed to the same cause, we need not be surprised to read that dysentery may be developed in the same way, etc., etc. With such a shortage in paper we wish this work had been reserved for after the war.

